# Clinical performance of additively manufactured subperiosteal implants: a systematic review

**DOI:** 10.1186/s40729-024-00521-6

**Published:** 2024-02-05

**Authors:** Eduardo Anitua, Asier Eguia, Christoph Staudigl, Mohammad Hamdan Alkhraisat

**Affiliations:** 1 University Institute for Regenerative Medicine and Oral Implantology, UIRMI (UPV/EHU-Fundación Eduardo Anitua), Jose Maria Cagigal Kalea, 19, 01007 Vitoria-Gasteiz, Araba Spain; 2 University of the Basque Country UPV/EHU and University Institute for Regenerative Medicine and Oral Implantology, UIRMI (UPV/EHU-Fundación Eduardo Anitua), Vitoria, Spain; 3 Department of Cranio-Maxillofacial Surgery, Kepler Universitätsklinikum, Linz, Austria; 4 BTI-Biotechnology Institute, Vitoria, Spain

**Keywords:** Subperiosteal implants, Custom-made implants, Maxillary atrophy, Implant survival

## Abstract

**Purpose:**

The aim of this study was to assess implant survival and complications rate of modern subperiosteal implants (CAD designed and additively manufactured).

**Methods:**

A systematic review was conducted using three electronic databases; Medline (Pubmed), Cochrane library, and SCOPUS, following the PRISMA statement recommendations to answer the PICO question: “In patients with bone atrophy (P), do additively manufactured subperiosteal implants (I), compared to subperiosteal implants manufactured following traditional approaches (c), present satisfactory implant survival and complication rates (O)? The study was pre-registered in PROSPERO (CRD42023424211). Included articles quality was assessed using the “NIH quality assessment tools”.

**Results:**

Thirteen articles were finally selected (5 cohort studies and 8 case series), including 227 patients (121 female / 106 male; weighted mean age 62.4 years) and 227 implants. After a weighted mean follow-up time of 21.4 months, 97.8% of implants were in function (5 failures reported), 58 implants (25.6%) presented partial exposure, 12 patients (5.3%) suffered soft tissue or persistent infection. Fracture of the interim prosthesis was reported in 8 of the155 patients (5.2%) in which the use of a provisional prosthesis was reported. A great heterogeneity was found in terms of study design and methodological aspects. For this reason, a quantitative analysis followed by meta-analysis was not possible.

**Conclusions:**

Within the limitations of this study, modern additively manufactured subperiosteal implants presented a good survival in the short-time, but a noticeable number of soft-tissue related complications were reported. Further studies are needed to assess the clinical behavior in the medium- and long-term.

**Graphical Abstract:**

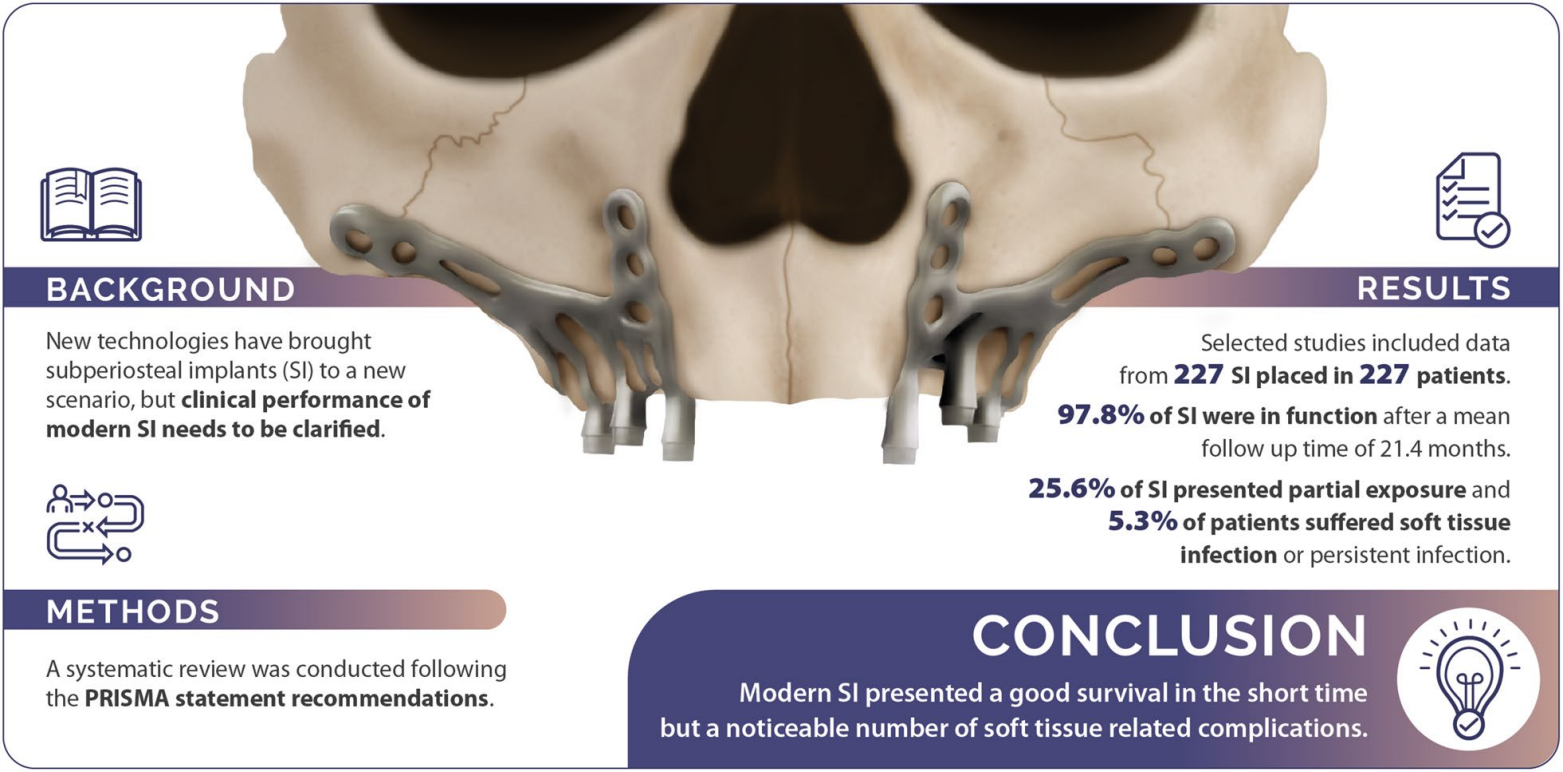

## Introduction

The use of subperiosteal implants (SI) was originally described by Dahl [1] in 1943, but gained relevance after the publication of Goldberg & Gerskoff [2] at the end of the 1940s. In the 1960s the basis of the so-called osseointegration was partially enlightened [3]. This scientific breakthrough, allowed implant dentistry to evolve from an experimental treatment to the current highly predictable option to replace missing teeth [3]. During this step-by-step transformation, SI have evolved like root-shape implants have drastically made. First SI were manufactured in Vitallium [4], (60% cobalt, 20% chromium, 5% molybdenum, and traces of other substances) [5] and were designed to support complete dentures (mostly removable). New SI designs were described and clinically assessed between the 1970s and the late 1990s. [6] At that time, acceptable 5-year results were documented (95% [7, 8] to 100% [9]) but long-term results regarding survival were less favorable (79% at 10 years [7], 76% at 10 years [8], 75% at 6 years [9], 67% at 10 years [10]). A less convenient for the patient two-time surgical approach was also required then. At the first surgery, a wide flap was raised to allow direct analogical bone surface impressions. During the second one, a casted Cr–Co alloy (or others) framework was adapted and placed beneath the mucoperiosteum without anchoring elements (such as osteosynthesis screws) in most of the cases. Lack of fitting and/or stability, unfavorable biomechanical design, and the use of unsuitable materials to achieve osseointegration, increased the risk of infection, implant exposition, and failure [4, 11]. In case of implant exposition, former SI designs impaired partial or full removal [12]. Probably, this further jeopardized both implant function and esthetics.

SI lost popularity among dental practitioners for a long period, but recent advances in computer-aided design (CAD), computer-aided manufacturing (CAM), the development of new materials (new Ti alloys or Polyether-ether-ketone or PEEK), improvements in surface treatments and a deeper understanding of bone biomechanical principles, have brought SI to a new scenario [13–18]. Thus, modern CAD designed and additively manufactured SI could provide advantages over former SI such as enabling one-time surgery and immediate loading, better fitting or surgical time reduction [14–16].

Frequently, clinicians must face the challenge of treating cases of severe bone atrophy or bone resection. Advances in root-shape implants design and size (short, extra-short and narrow implants) have provided new solutions or have enhanced older ones, for the treatment of different types of bone atrophy [19–21]. Different accessory surgical techniques for recovering the lost bone volume were also developed and improved to treat those patients where root-shape implants could not be placed directly [22–26]. Among them, guided bone regeneration (GBR), maxillary sinus and nasal floor augmentation, inlay or onlay bone grafting, distraction osteogenesis, nerve lateralization or others have been routinely employed with a varying degree of clinical success [22–26]. The use of zygomatic implants could be also a reliable option for the treatment of those patients with severe posterior maxillary atrophy [27]. The success rate and the incidence and severity of postoperative complications using this type of implants is dependent of clinician expertise [28].

Modern SI have been claimed to present some advantages to treat certain patients with bone atrophy over the above-mentioned techniques. The elimination of bone donor area morbidity (in the case of autologous bone grafting need), the possibility of ambulatory realization and reduction of surgical time, are among the reported benefits for patients [14]. Modern SI also provide an option of treatment for patients with extreme bone defects due to oncologic disease treatment or trauma [29, 30]. On the other hand, the digital resources (devices and software) required to design and manufacture SI are not accessible to all professionals, and the clinical performance of SI is still not well evidenced. This systematic review attempts to assess the clinical performance of modern additively manufactured SI by analyzing their survival and complications rate data available in the literature.

## Materials and methods

A systematic review was carried out following the Preferred Reporting Items for Systematic Reviews and Meta-Analyses (PRISMA) statement recommendations [31] to answer the following the PICO questions: “In patients with bone atrophy (P), do additively manufactured subperiosteal implants (I), compared to subperiosteal implants manufactured following traditional approaches (c), present satisfactory implant survival and complication rates(O)?”. The aim of this review was to answer with the best available evidence, this question to help clinicians when planning the treatment of patients with maxillary or mandibular bone atrophy.

### Protocol and registration

A register in the International Prospective Register of Systematic Reviews (PROSPERO) of the National Institute for Health Research (NIHR) was obtained before starting (CRD42023424211). The PRISMA guidelines for systematic reviews were used to conduct the review process [31].

### Eligibility criteria, information sources and search

Three electronic databases including Medline, Cochrane library, and Scopus were searched. To build the search strategy (PICO), the following considerations were applied:

- *Patient* Patients presenting maxillary or mandibular bone atrophy in the need for oral rehabilitation.
- *Intervention* CAD designed and additively manufactured subperiosteal implant placement (1-time surgical approach).
- *Comparison* Traditional custom-made subperiosteal implants (manufacture of the framework by casting methods; direct impression of bone surface; 2-time surgical approach).
- *Outcomes* Implant survival. Complications rate.

The main question built was then as follows: “In patients with bone atrophy, do additively manufactured subperiosteal implants, compared to subperiosteal implants manufactured following traditional approaches, present satisfactory implant survival and complication rates?”. In the search strategy (Table 1), following terms were employed: “dental implantation, subperiosteal” (MeSH Term) “subperiosteal implant(s)” (free term) and “juxta-osseous implants” (free term). The search query was generated as follows: “dental implantation, subperiosteal”[MeSH Terms] OR “Subperiosteal implant”[All Fields] OR “subperiosteal implants”[All Fields] OR “juxta-osseous implants”[All Fields].

**Table 1:** Summary of the search strategy followed to select the articles included in the qualitative synthesis

Search strategy Databases: Medline, Scopus, Cochrane Library Date: 11/5/2023 Language: English, Spanish Time limits: No Search terms: subperiosteal implants, juxta-osseous implants
PICO strategy: *In patients with severe bone atrophy (P), do additively manufactured subperiosteal implants (I), compared to subperiosteal implants manufactured following traditional approaches (c), present satisfactory implant survival and complication rates(O)?*

	Database searched	Search strategy	#Records	#Duplicates	#Excluded after screening		#Records included
Identification	Pubmed-Medline	Search 1: “dental implantation, subperiosteal”[MeSH Terms] OR “Subperiosteal implant”[All Fields] OR “subperiosteal implants”[All Fields] OR “juxta-osseous implants”[All Fields]	389	–	308		11
	SCOPUS	Search 1: “subperiosteal implants” OR “subperiosteal implant” OR “juxta-osseous implants”	383	1	382		0
	Cochrane Library	Search 1: “subperiosteal implants” Search 2: “subperiosteal implant” Search 3: “juxta-osseous implants”	20	0	20		0
	Other sources						
	Manual search (including Free terms) in the same databases						8
	Citation searching (references of included studies)						2
	Internet						6
	Grey literature (University of London Online Library, Worldcat, Open Grey, WorldWideScience.org)						4
	Total						812
Screening	Records excluded and reasons - Duplicates - Not focused on the review topic - No clinical studies - Study design: not in accordance with inclusion criteria					Total records excluded: 795	
Eligibility	Full-text articles excluded with reasons	- Van den Borre C et al. Radiographic Evaluation of Bone Remodeling after Additively Manufactured Subperiosteal Jaw Implantation (AMSJI) in the Maxilla: A One-Year Follow-Up Study. J Clin Med. 2021 Aug 12;10(16):3542.^a,c^ - Elsawy MA, et al. Polyetheretherketone subperiosteal implant retaining a maxillary fixed prosthesis: A case series. J Prosthet Dent. 2022 Oct 6:S0022-3913(22)00554–6.^b^ - Mommaerts MY. Evolutionary steps in the design and biofunctionalization of the additively manufactured sub-periosteal jaw implant ‘AMSJI’ for the maxilla. Int J Oral Maxillofac Surg. 2019 Jan;48(1):108–114.^a^ - Jehn P, Spalthoff S, Korn P, Stoetzer M, Gercken M, Gellrich NC, Rahlf B. Oral health-related quality of life in tumour patients treated with patient-specific dental implants. Int J Oral Maxillofac Surg. 2020 Aug;49(8):1067–1072.^a^				Total records excluded:4	
		Reasons					
		^a^No information available to answer the PICO question					
		^b^Exclusion criteria: Only Implants additively manufactured					
		^c^Same patient series as in another already included study					
Included		Studies included in qualitative synthesis				13	

This electronic search was complemented by:

- Review of the full-text selected articles reference lists.
- Manual searches in the same databases including other free terms such as “custom-made implants”, “Direct Metal Laser Sintering”, “patient-specific implants” or “additively manufactured implants”.
- Grey literature (University of London Online Library, Worldcat, Open Grey, WorldWideScience.org)
- Internet free search.

No restrictions of time were applied. Only articles in English or Spanish were assessed for eligibility. Two authors independently assessed the publications by title and abstract. The inclusion or exclusion criteria for the studies were as follows:

1. *Inclusion criteria*:
2. Clinical studies in humans: Randomized Clinical Trials (RCTs), prospective and retrospective cohort or case–control studies, and case series.
3. Subperiosteal implants CAD designed and additively manufactured.
4. *Exclusion criteria*:
5. Case reports.
6. Studies without information related to the measured outcomes.

### Study selection

The study selection was performed by the same two independent reviewers and an additional reviewer acted in case of disagreement. After article selection based on the abstract and the article selection criteria, both reviewers read the complete articles and determined whether they met the inclusion criteria for this review. Agreement in the selection process was calculated using Cohen’s kappa coefficient, with a κ value of 0.81 (92.31% of agreement).

### Data collection process

Data from all articles were collected in duplicate by both researchers independently and then pooled in the same worksheet. The following information was extracted from each selected study: year of publication, type of study, number of patients and implants, sex and age of patients, cause of bone defect, inclusion criteria, implant material, manufacturing technology, implant location, design and surface, type of bone fixation, type of prosthetic rehabilitation and retention system, usage of interim prosthesis, design and materials of definitive prosthesis, surgery time, implant fitting rating, follow-up, implant survival, and complications.

### Data synthesis and outcomes

Data from the identified and relevant publications were extracted and, if indicated, presented in evidence tables. The main outcomes analyzed were:

- *Implant survival.* Defined as the presence of the implant in function in the mouth after the end of the follow-up period established in each study.
- *Complications.* Including technical complications affecting both the implant or the prosthesis and all type of biological complication affecting the bone or soft tissues.

### Risk of bias in individual studies

The methodological quality of the included studies was assessed using The National Institutes of Health—”NIH quality assessment tools’’ for case series and for observational Cohort and Cross-Sectional Studies. Although “NIH quality assessment tools” were initially conceived to help reviewers, these tools have been broadly used in many recent systematic reviews to assess the study quality [32, 33]. The risk of bias was measured independently by two authors, and in cases of disagreement, a third author participated to solve it.

### Summary measures

All the variables were collected in a database and analyzed with IBM SPSS statistics v. 20.0 (IBM Corp., Armonk—NY, USA). For the univariate description, we employed basic descriptive statistics.

## Results

### Study selection

The initial search provided 792 articles. Additional searches allowed to identify 20 more articles. Before Screening 612 articles were removed. Additionally, 183 articles were also removed after the abstract review. Twelve articles were assessed for eligibility, but after a deep analysis of the article, 4 were excluded for the following reasons:

- No information available to answer the PICO question (*n* = 1) [30, 34].
- Not complying with inclusion criteria: Implants additively manufactured (*n* = 1) [35].
- Same patient series as in another already included study [36] and no information available to answer the PICO question (*n* = 1) [37].

**Fig. 1:**
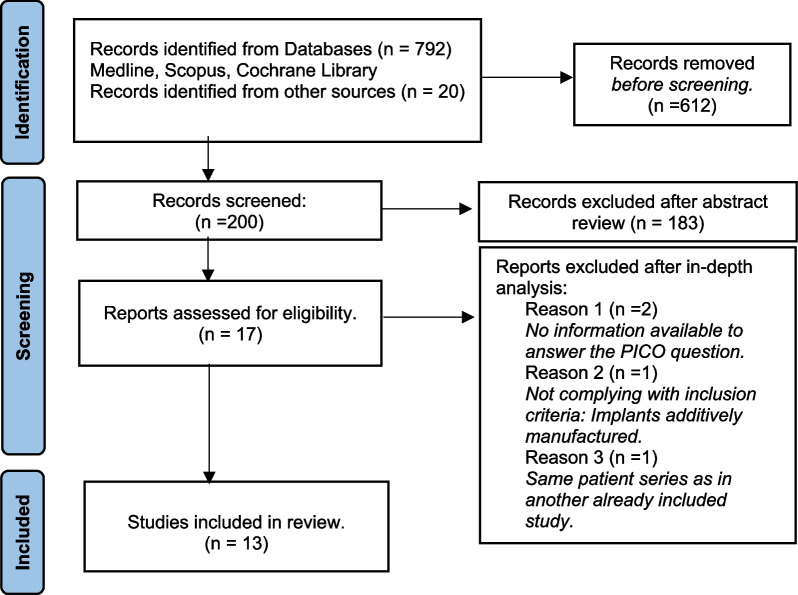
Search strategy flow. Adapted from The PRISMA 2020 statement (Page et al. [38])

Figure 1 summarizes the study selection process in a *Flow Diagram* adapted from Page et al. [38].

### Study characteristics

The 13 articles finally included in the review [29, 36, 39–49] corresponded to 5 cohort studies (1/5 prospective, 4/5 multicentric) and 8 case series, (1/8 multicentric), that involved a total of 227 patients and the same number of unilateral/bilateral, maxillary/mandibular implants. No RCTs or previous systematic reviews were found during literature search. All the included articles had been published from 2017 onwards.

### Risk of bias within studies

Two articles were multicentric and performed by the same International Group of authors [36, 39] at nearby dates. Possible patient overlapping among both articles is unknown.

### Synthesis of results

The finally selected studies included data from 227 SI placed in 227 patients (121 female / 106 male) with a weighted mean age of 62.4 years. The location of the SI was specified only for 162 implants (142 maxilla / 20 mandible) and not clearly stated for 65 SI.

The main reason for implantation was bone atrophy. A Cawood–Howell atrophy type V or higher was an inclusion criteria in 5/13 studies, including 94/227 (41.5%) SI. In 24 patients (10.6%) it was clarified that a resective/maxillectomy had been previously performed. One hundred and fourteen patients (50.2%) required a full-arch rehabilitation, 29 (12.8%) a partial restoration and in 84 patients (37%) the type of rehabilitation was not specified (Table 2).

**Table 2:** Summary of included studies; study type, demographic data from included patients and inclusion criteria

Authors	Year	Type of study	*n* (patients)	Mean age. (years)	Sex	*n* (implants)	Cause of bone defect	Inclusion criteria
Mangano et al. [40]	2020	Case series	10	69.6 range: 68 to 75	6F/4 M	10	Bone atrophy	Healthy patients Age > 65 years Nonsmoker Partially edentulous (≥ 2 teeth) Residual bone < 10 mm Acceptable oral hygiene Regenerative bone surgery unwillingness
Van den Borre et al. [36]	2022	Prospective Multicenter Study	15	Male: 57.4; SD ± 8.7 Female: 62.2; SD ± 3.4	7F/8 M	15	Bone atrophy	Cawood–Howell atrophy ≥ 5 Consecutive patients Bilateral placement in the maxilla
Van den Borre et al. [39]	2023	Retrospective Multicenter Study	40	Male: 64.6; SD ± 6.7 Female: 65.2; SD ± 6.8	25F/15 M	40	Maxillary defect reconstructions were excluded	Placement at least one year before assessment 122 patients eligible; definitive inclusion by patient and surgeon decisions Maxillary severe atrophy (Cawood–Howell atrophy ≥ 5)
Chamorro Pons et al. [41]	2021	Case series	8	72.2	6F/2 M	8	Bone atrophy	Cawood–Howell atrophy ≥ 5 No systemic contraindications
Cebrián et al. [29]	2022	Case series	4	66.2	3F/1 M	4	Segmental maxillary oncological defect (due to OSCC)	Patients with segmental maxillary oncological defect that had been reconstructed with a subperiosteal implant
Nemtoi et al. [42]	2022	Retrospective Cohort Multicenter Study	16	61.5 Range: 55 to 69	7F/9 M	16	Bone atrophy	Patient over the age of 55 years Treated with DMLS manufactured subperiosteal implant Equilibrated general and oral health Improved oral hygiene Nonsmoker Available bone height ≤ 10 mm Regenerative bone surgery unwillingness
Cerea et al. [43]	2018	Retrospective Multicenter Study	70	62.8 Range: 62 to 79	31F/39 M	70	Bone atrophy	Patient over the age of 60 years Treated with DMLS manufactured subperiosteal implant 2-year minimum follow-up Nonsmoker and not bruxist
Dimitroulis et al. [44]	2023	Case series Multicenter Study	21	59.1 Range: 31 to 80	14F/7 M	21	Bone atrophy. Maxillectomy (1/21)	Cawood–Howell atrophy ≥ 5 Partial or fully edentulous arches Nonsmoker Not suffering from a terminal ill or severe medical conditions (as radiotherapy of the jaws)
Mounir et al. [45]	2018	Observational clinical study	5*	27.4 Range: 18 to 55	1F/4 M	5	Bone atrophy	Anterior maxillary bone defect Not enough bone volume to room standard root-form implants (with at least 3 mm of diameter and 8 mm height) No systemic disease or oral pathosis that may affect bone healing No previous grafting procedure at the implant site
Gellrich et al. [46]	2017	Case series	3	68 Range: 55 to 90	2F/1 M	4	Bone atrophy (2/3 due to OSCC)	History of severe bone loss Patients requesting implant-supported dental prosthesis in the absence or impossibility of bone reconstruction by conventional techniques No history of bisphosphonate therapy, psychiatric disorder, alcohol-related diseases, or active smoking
Korn et al. [47]	2022	Case series	10	66 Range: 50 to 90	7F/3 M	13 (3 bilateral)	Bone atrophy	Cawood–Howell atrophy ≥ 5 No head-neck cancer history or previous irradiation No cleft lip or palate, or trauma history
Rahlf et al. [48]	2022	Case series	6	51 Range: 18 to 68	3F/3 M	6	Cleft lip and palate deformity (CLP)	CLP-associated deformity Maxillary partial or total edentulism
Korn et al. [49]	2021	Case series Single-center Study	19	65 Range: 30 to 85	9F/10 M	20 (1 bilateral)	Bone atrophy. 15/20 due to oral malignancy treatment 5/20 due to aggressive oral lesion treatment	Previous tumor resection No history of failed augmentation procedure, trauma, or cleft palate

*This study included two groups of implants. Group 1 (5/10 Ti implants) was included. Group 2 1 (5/10 PEEK implants) was excluded

Different implant designs were used across different studies, but in all cases a variable number of osteosynthesis screws were employed to anchor the framework to the bone. All the SI were manufactured in Ti alloys. In the study of Mounir et al. [45], 5 implants included in the group 2 were manufactured in polyether-ether-ketone (PEEK). Data from this 5 PEEK implants were excluded.

The surface in contact with the bone was porous (rough) in 5 studies (83 implants), polished (electroerosion) in 1 study (4 Implants) and not clearly stated in 7 studies (140 implants) (Table 3).

**Table 3:** Summary of subperiosteal implants location, characteristics, design, and manufacture

Authors	Implant material	Manufacturing technique	Implant location (maxilla/mandible)	Implant design	Implant surface (bone face)	Implant fixation
Mangano et al. [40]	Ti grade V	DMLS (direct metal laser sintering)	Posterior mandible	Buccal and lingual arms for implant fixation. Tapered posts for prosthetic cementation	Porous	Osteosynthesis screws Buccal and lingual
Van den Borre et al. [36]	Ti grade 23	Additive manufacture (technique not specified in the text)	Maxilla	2-piece implants (bilateral) splinted by the prostheses. On each piece: Fixation vestibular arms (2), prosthetic connecting posts (3)	Porous	Osteosynthesis screws Buccal arms
Van den Borre et al. [39]	Ti grade 23	Additive manufacture (technique not specified in the text)	Maxilla	2-piece implants (bilateral) splinted by the prostheses. On each piece: Fixation vestibular arms (2), prosthetic connecting posts (3)	Porous	Osteosynthesis screws Buccal arms
Chamorro Pons et al. [41]	Ti	Additive manufacture (technique not specified in the text). Micro-milled connections	Maxilla	Bilateral main structure with detachable arms (2 or 3 pieces). 6 prosthetic connecting posts (external hexagonal or conical connection). 0.8 mm maximum thickness	N.A	14 to 16 osteosynthesis screws (Ø1.5 mm)
Cebrián et al. [29]	Ti	Sinterization	Maxilla	Titanium mesh/plate and prosthetic connecting posts (4 or 6). External hexagonal connection (universal, 4.1 mm)	N.A	Osteosynthesis screws
Nemtoi et al. [42]	Ti	DMLS (direct metal laser sintering). Post-mechanization	11/16 Maxilla; 5/16 Mandible	0.7 mm thickness. Arms for fixation with osteosynthesis screws	Rough	Osteosynthesis screws
Cerea et al. [43]	Ti grade V	DMLS (direct metal laser sintering)	Maxilla or mandible (no further information available)	Buccal and lingual arms for implant fixation. Tapered posts for prosthetic cementation	Polished (electroerosion)	Osteosynthesis screws
Dimitroulis et al. [44]	Ti	Laser sintering	18/21 Maxilla; 3/21 Mandible	Buccal and lingual arms for implant fixation. At least 8 screws placed buccally and additionally, 2 or more in lingual/palatal position. Tapered posts	N.A	Osteosynthesis screws (Ø2 mm in the mandible and Ø1.6 mm in the maxilla)
Mounir et al. [45]	Ti grade 23 (group 1)	EBM (electron beam melting)	Anterior maxilla	Buccal plate/mesh, buccal holes for the osteosynthesis screws (Ti implants meshed with 2.3 mm holes) and cylindric posts (3 to 6) for prosthetic connection (cemented)	Rough (acid-etching)	Osteosynthesis screws (Ø2 mm)
Gellrich et al. [46]	Ti grade 23	SLM (selective laser melting)	Maxilla (1/4), Mandible (3/4)	Anchorage framework with holes for multiple osteosynthesis screw. Prosthetic posts for internal conventional implant connection, ball attachment system or external conical (telescopic) crow connection	N.A	Osteosynthesis screws
Korn et al. [47]	Ti grade 23	SLM	Maxilla	Anchorage framework with holes for multiple osteosynthesis screw. Four connection posts	N.A	Osteosynthesis screws (Ø1.5–2 mm)
Rahlf et al. [48]	Ti grade 4	SLM	Maxilla	Anchorage framework with holes for multiple osteosynthesis screw. Two to four connection posts	N.A	Osteosynthesis screws (Ø1.5 mm)
Korn et al. [49]	Ti grade 23	SLM	Maxilla	Anchorage framework with holes for multiple osteosynthesis screw. Two to four connection posts	N.A	Osteosynthesis screws (Ø1.2–2 mm)

Regarding prosthetic rehabilitation, 113 SI supported a fixed denture, while in 114 patients in was not specified the actual number of fixed and removable dentures. In 7/13 studies, 144 implants were loaded with interim prostheses at different times after surgery. Prosthetic connection was screw-retained for 104 SI (45.8%) and cemented for 90 SI. For screw-retained restoration, the most common number of connecting posts was 6 (63/104). Definitive prostheses were highly variable in terms of manufacturing techniques, materials, and time of loading (Table 4).

**Table 4:** Prosthetic rehabilitation; characteristics of temporary and definitive prosthesis

Authors	Type of rehabilitation (partial / full-arch)	Type of prosthesis (fixed / removable)	Provisional prosthesis (use & features)	Prosthesis fixation	Prosthesis impression technique	Definitive prosthesis
Mangano et al. [40]	Partial	Fixed	Yes 2 sets Milled in PMMA	Cemented. Temporary cement	Digital Intraoral scanner	Zr framework Delivered after 2 months
Van den Borre et al. [36]	Full-arch	Both	Yes Additively manufactured	Screw-retained. 6 connecting posts	N.A	Overdenture with connecting bar or hybrid FCD Delivered after 2 months
Van den Borre et al. [39]	Full-arch	Both	N.A	Screw-retained. 6 connecting posts	N.A	Fixed or removable (no further information available)
Chamorro Pons et al. [41]	Full-arch	Fixed	Yes. Premanufactured acrylic prostheses with holes to bond it to temporary abutments. Screw-retained	Screw-retained. 6 connecting posts	N.A	6/8 Metal (Cr–Co) CAD/CAM suprastructure veneered with porcelain 2/8 Resin hybrid prostheses Delivered after 1.5 to 2 months
Cebrián et al. [29]	Full-arch	Fixed	Yes. Two weeks after surgery	Screw-retained. 4 or 6 connecting posts	Analogical (open tray)	Metal () CAD/CAM suprastructure veneered with porcelain. Delivered after 2 months
Nemtoi et al. [42]	14/16 full-arch 2/16 partial	N.A	Yes. Within 12 h. After surgery. Fixed acrylic resin prosthesis	Screw-retained	N.A	After 6 months (no further information available)
Cerea et al. [43]	Full-arch or partial	Fixed	Yes. Fixed acrylic resin prosthesis. Within 48 h after surgery	Cemented	Analogical (polyvinylsiloxane)	CAD/CAM metallic suprastructure veneered in ceramic. Delivered after 3–4 months
Dimitroulis et al. [44]	18/21 Full-arch; 3/21 partial (maxillary)	Fixed	Yes. (15/21) CAD/CAM Ti suprastructure and cemented acrylic overlay. Both manufactured using milling methods	Screw-retained	N.A	Delivered after 2 to 6 months
Mounir et al. [45]	Partial	Fixed	Acrylic bridges. delivered after 1 month at least No further information available	Cemented	N.A	Delivered after 1 month at least No further information available
Gellrich et al. [46]	2/3 Partial, 1/3 Full-arch	Removable	No provisional prosthesis	–	Analogical (polyether)	Delivered after 3–4 months
Korn et al. [47]	Full-arch	N.A	N.A	–	Analogical/digital	N.A. Only 11/14 implants loaded
Rahlf et al. [48]	2/6 Partial, 4/6 Full-arch	Removable	1/6 provisional prosthesis	–	N.A	N.A. 5/6 implants loaded
Korn et al. [49]	N.A	Both	N.A	–	N.A	N.A

### Complication rate of SI

After a weighted mean follow-up time of 21.4 months (mean range 1 to 74 months), 97.8% of implants were in function (5 failures reported). In 3 studies [29, 36] (including 22 patients), no complications were reported. Post-operative complications (pain, discomfort, bleeding, swelling) was reported in 17 patients (7.5%), 58 implants (25.6%) presented partial exposure, 12 patients (5.3%) suffered soft tissue infection or persistent infection. The use of a provisional prosthesis was reported in 155 patients. Fracture of the interim prosthesis was reported in 8/155 patients (5.2%). Implant fitting during surgery was assessed in 4 studies [40–42, 44] including 55 SI and rated as satisfactory in 48/55 (87.7%) of the assessed implants (Table 5).

**Table 5:** Follow-up time and summary of clinical outcomes

Authors	Mean surgery time (min)	Follow-up (months)	Implant survival	Implant fitting	Complications
Mangano et al. [40]	44.3 ± SD 19.4	12	100%	Mean rating: 7 out of 10 SD ± 1.6, median 7, 95% CI 6–8 Satisfactory 8/10 Insufficient 2/10* *adapted during surgery and placed	1/10 patient immediate postoperative complications (pain, discomfort, swelling) 2/10 patient late complications (provisional restoration fracture)
Van den Borre et al. [36]	N.A	12	100%	N.A	No complications reported
Van den Borre et al. [39]	N.A	30.1 *917 days; SD ± 306.89 days	100%	N.A	12/40 postoperative inflammation (i.e., swelling, marked redness, pain) 6/40 apparent soft tissue infection, drainage, exploration and/or mechanical debridement needed 3/40 required one connecting post removal due to persistent and uncontrollable infection 26/40 Partial exposure of the arms not experienced as a functional or esthetic impediment by patients 1/40 Mobility of the implant (> 1 mm)
Chamorro Pons et al. [41]	80	mean: 18.4 range: 4 to 36	100%	Satisfactory 8/8	1/8 needed prosthetic removal and recontouring (soft tissue inflammation/ulceration)
Cebrián et al. [29]	N.A	mean: 20 range: 9 to 38	100%	N.A	No complications reported
Nemtoi et al. [42]	86	12	93%	5/16 not fully satisfactory Mean satisfaction rate: 4/5	3/16 bleeding 6/16 implant exposure 1/16 implant failure 1/16 fracture of temporary prosthesis
Cerea et al. [43]	N.A	24	95.8%	N.A	3/70 failure due to infection 4/70 postoperative pain/discomfort/swelling 1/70 recurrent infections 4/70 fracture of provisional prosthesis 2/70 ceramic chipping in the definitive prosthesis
Dimitroulis et al. [44]	N.A	Mean: 22.1 range: 5 to 57	95% (85.7% success rate)	Satisfactory 21/21	1/21 Failure (explanted because of chronic pain) 4/21 Salvaged (replacing exposed frames or adding more bone screws) 2/21 (considered failures because exposure of the framework even though the device is still functional)
Mounir et al. [45]	N.A	12	100%	N.A	1/5 wound dehiscence and exposure of the implant. Fully covered subsequently after removal of uncovered rim of the implant 5/5 Ti implants showed 1–2 mm exposure of the platform around the posts. (No interference with prosthetic loading or patient dissatisfaction was reported)
Gellrich et al. [46]	N.A	Mean: 18 range: 14 to 21	100%	Satisfactory 3/3	No complications reported except for partial discomfort/pain in one patient
Korn et al. [47]	135	Mean: 8.2 range: 1 to 29	100%	N.A	Infection 1/10 patients Exposure of the framework 2/10 patients Screw-loss 1/10 patients
Rahlf et al. [48]	146	Mean:18.2 range: 6 to 40	100%	N.A	6/6 chronic mucositis 3/6 Framework exposure around posts
Korn et al. [49]	127	Mean: 26 Range: 6 to 74	100%	N.A	1/20 severe infection 1/20 exposed screws needed remotion 9/20 Exposure of the framework

### Risk of bias across studies

Individual study Quality assessment was performed using the NIH—Study Quality Assessment Tool for case series and for cohort studies. Two articles were rated as “Poor”, 2 as “Good” and as “Fair” (Tables 6, 7). Due to the type of study design in selected studies and the great heterogeneity found in methodological aspects, a quantitative analysis followed by meta-analysis was not possible.

**Table 6:** Quality assessment of included articles: *Cohort studies*: (1) Was the research question or objective in this paper clearly stated? (2) Was the study population clearly specified and defined? (3) Was the participation rate of eligible persons at least 50%? (4) Were all the subjects selected or recruited from the same or similar populations (including the same time period)? Were inclusion and exclusion criteria for being in the study prespecified and applied uniformly to all participants? (5) Was a sample size justification, power description, or variance and effect estimates provided? (6) For the analyses in this paper, were the exposure(s) of interest measured prior to the outcome(s) being measured? (7) Was the timeframe sufficient so that one could reasonably expect to see an association between exposure and outcome if it existed? (8) For exposures that can vary in amount or level, did the study examine different levels of the exposure as related to the outcome (e.g., categories of exposure, or exposure measured as continuous variable)? (9) Were the exposure measures (independent variables) clearly defined, valid, reliable, and implemented consistently across all study participants? (10) Was the exposure(s) assessed more than once over time? (11) Were the outcome measures (dependent variables) clearly defined, valid, reliable, and implemented consistently across all study participants? (12) Were the outcome assessors blinded to the exposure status of participants? (13) Was loss to follow-up after baseline 20% or less? (14) Were key potential confounding variables measured and adjusted statistically for their impact on the relationship between exposure(s) and outcome(s)?

NIH quality assessment tool for observational cohort and cross-sectional																
Authors	Study type	1	2	3	4	5	6	7	8	9	10	11	12	13	14	Rating
Van den Borre et al. [36]	Prospective multicenter study	*	*	–	*	–	*	*	^o^	*	^o^	*	^o^	–	–	Fair
Van den Borre et al. [39]	Retrospective multicenter study	*	*	–	*	–	*	*	^o^	*	^o^	*	^o^	^o^	–	Fair
Nemtoi et al. [42]	Retrospective cohort multicenter study	*	*	*	*	–	^o^	*	^o^	*	^o^	*	^o^	^o^	–	Fair
Cerea et al. [43]	Retrospective multicenter study	*	*	^o^	*	–	^o^	*	^o^	*	^o^	*	^o^	^o^	–	Fair
Mounir et al. [45]	Observational clinical study	*	*	^o^	*	–	^o^	*	^o^	*	^o^	*	^o^	^o^	–	Fair

*Yes

–No

^o^N.A.: not applicable / N.R.: not disclosed

**Table 7:** Quality assessment of included articles: *Case series*: (1) Was the study question or objective clearly stated? (2) Was the study population clearly and fully described, including a case definition? (3) Were the cases consecutive? (4) Were the subjects comparable? (5) Was the intervention clearly described? (6) Were the outcome measures clearly defined, valid, reliable, and implemented consistently across all study participants? (7) Was the length of follow-up adequate? (8) Were the statistical methods well-described? (9) Were the results well-described?

NIH quality assessment tool for case series studies											
Author	Study type	1	2	3	4	5	6	7	8	9	Rating
Mangano et al. [40]	Case series	*	*	^o^	*	*	*	*	*	*	Good
Chamorro Pons et al. [41]	Case series	*	*	*	*	*	–	–	–	*	Fair
Cebrián et al. [29]	Case series	*	*	–	*	*	–	–	–	*	Fair
Dimitroulis et al. [44]	Case series multicenter study	*	*	–	*	*	*	*	–	*	Good
Gellrich et al. [45]	Case series	**–**	*	–	**–**	**–**	*	**–**	–	*	Poor
Korn et al. [46]	Case series	**–**	*	–	**–**	**–**	*	**–**	–	*	Poor
Rahlf et al. [47]	Case series	*	*	–	**–**	**–**	*	*	–	*	Fair
Korn et al. [48]	Case series	*	*	–	*	**–**	*	*	–	**–**	Fair

*Yes

–No

^o^N.A.: not applicable / N.R.: not disclosed

### Strength of evidence (SoE)

In absence of randomized studies, the level of evidence was initially rated as “Low”, attending GRADE (Grading of Recommendations, Assessment, Development and Evaluations) system [50]. After assessment of domains that could rate down (Risk of bias, Imprecision, Inconsistency, Indirectness and Publication bias) or rate up (Large magnitude of effect, Dose–Response gradient, Confounding factors) the SoE evaluation was downrated to “Very Low”.

## Discussion

CAD designed additively manufactured SI presented satisfactory survival (97.8%) in the short-term (weighted mean follow-up time 21.4 months; mean range 1 to 74 months), but there is a paucity of data on their success rates and medium- or long-term clinical behavior. Available data are coming only from observational studies (cohort studies and case series), including 227 unilateral/bilateral implants (in 227 patients). From them, 70/227 (31%) came from the same retrospective study [43] and 55/227 (24%) came from two multi-center studies using same type of implant and performed by the same international team [36, 39]. Another 35/227 (15.4%) came from 3 single-center studies including patients treated with the same type of implant at the same center [47–49].

The most frequent complications reported are those related to soft tissues. Hereby, partial exposure of the framework seems to be the most frequent complication, although this seems not to conditionate the survival in the short-term. New designs could allow to remove exposed parts or prosthetic posts in an easier and safer way than in former designs [13, 39, 44]. Although this fact has not yet been specifically evidenced in the literature, this improvement could positively influence the success of modern SI.

From those patients where the use of a provisional prosthesis was stated, 5.2% suffered a fracture of the interim prosthesis. Despite no further information is available to analyze the reasons, to ensure a good passive fitting of the prosthesis, to carefully adjust the occlusion and to reinforce the framework of interim prosthesis seems advisable for these patients as it is for those wearing conventional root-shape implants [51, 52].

Although the location of the implant was not specified in 65 patients [43], there was a noticeably higher number of maxillary than mandibular implants (142:20). Furthermore, 93/142 (65.4%) of maxillary implants had been manufactured following the same two specific design concept, and material [36, 39, 46–49]. From those stated to have been placed in the mandible (20), 11 supported partial rehabilitations, so the extrapolation of the results of this review to full-arch mandible SI must be very prudently performed.

Bone implant fitting during surgery (in the 4 assessed studies) [39–42, 44] was satisfactory in mostly all cases. However, it was only assessed in 55 implants and the way of rating this outcome was based on personal feedback and potentially subjective. In those patients where fitting was unsatisfactory, the time of surgery was increased to make the implant fit properly to the bone contour. Dimitroulis et al. [44] noted that longer time (> 3 months) between the CT scan and delivery of the SI (what could cause further bone remodeling) or CT slices greater than 1 mm (which reduced the accuracy and tolerance of the device) could influence misfitting.

The main reason for implantation in the selected studies was bone atrophy. A Cawood–Howell atrophy type V or higher was an inclusion criteria in 5/13 studies [36, 39, 41, 44, 47], including 94/227 (41.5%) SI. In 24 patients (10.6%) it was clarified that a resective/maxillectomy had been previously performed. The studies from Mangano et al. [40] and Nemtoi et al. [42] included patients with a residual bone < 10 mm and regenerative bone surgery unwillingness on the part of the patient. In these two last studies the advantages and disadvantages of SI over the use of extra-short implants (≤ 6.5 mm) without the need for ancillary bone regenerative procedures could be arguable in the absence of more specific information about each specific case. Extra-short root-shape implants have evidenced in recent systematic reviews and meta-analysis, similar clinical performance to standard-length ones in terms of marginal bone loss (MBL), technical complications or implant survival [52–57]. The possibility of placing an immediate prosthesis, the peculiarities of the type of bone defect in each specific case or the experience degree of the surgeons may have influenced this decision, although the real reasons are unclear. The same can be argued to the study from Mounir et al. [45] where an inclusion criteria was enough bone volume to room standard root-shape implants with at least 3 mm of diameter and 8 mm of length.

Despite SI have a long history, their use is secondary to the use of endo-osseous root-shape implants, both in terms of experience and evidence. Until two decades ago, they were mainly used to support mandibular full-arch removable prostheses [4, 7–9, 58]. In oldest designs, SI were not directly anchored to the bone with osteosynthesis screws or other systems (to avoid movement), were manufactured by casting, required a two-time surgical procedure (first one to take direct impressions of the bone) and good bone–implant fitting was complex to achieve [4, 6–11]. Studies with these oldest designs and materials showed poor clinical results in the medium- or long-term [6]. Between the 1980s and the 1990s, success rates at 5-year ranging from 90% [10] to 100% [9] were reported but survival rates decreased at 6 years (75%) [10], 10 years (87%) [59] or 13 years (78%) [59]. Furthermore, most of articles did not include other results (in addition to implant survival) that would allow a reliable assessment of the success rate or the degree of patient satisfaction. Considering studies from 90s onwards, a 10-year survival rate of 79% was reported by Yanase et al. [7] and 76% by Bodine et al. [8] A 6-year evaluation performed by Ferrer et al. [60] revealed a 92.5% success of SI including design innovations and an 84% success for SI with classical designs.

Aforementioned paucity of data does not allow to compare medium- or long-term clinical behavior of modern additively manufactured SI and former ones. In any case, several improvements have been incorporated that could be helpful to improve survival, success and/or patient patient´s satisfaction degree, but this is yet to be evidenced. Among these improvements, a better understanding of the of biomechanics trough finite elements studies has allowed to reduce stress accumulation on bones, implants, abutments, and prosthetic frameworks [61, 62]. Golec [63] anticipated in 1986 the use of CAD/CAM to eliminate the need for surgical bone impression. Since then, several improvements in CBCT definition and additive manufacture refinement were needed to obtain more precise frameworks (reducing misfitting and/or micromovements) [34, 64, 65]. Surface features are also involved in the optimization of SI–bone surface interactions. A higher number of the implants included in this revision were porous (rough) on the bony face to promote osteointegration, and smooth (polish) on the soft tissue face to prevent biofilm colonization [13, 34, 66]. Modern manufacturing and new materials resistance allowed to reduce the thickness of the framework up to 0.7- or 0.8-mm [41, 42]. Further than weight lightening, this reduction seems helpful to prevent exposition. On the other hand, small connections also may lead to more fractures, although the limits of thinning are yet to be studied in more depth.

In relation to SI design too, 4–6 prosthetic posts (implant-prosthesis connectors) were preferred in most studies. All these improvements could have contributed to maintain bone and soft tissue stability. In this sense, Van den Borre et al. [37] performed a radiographic evaluation of modern SI and observed acceptable bone remodeling in the underlying bone (mean negative bone remodeling over six reference points on the crest: 0.26 mm ± SD 0.65 mm; mean bone remodeling at the supporting bone at the wings and basal frame: 0.088 mm ± SD 0.29 mm).

No differences in clinical performance between cemented and screw-retained fixed prostheses could be demonstrated. This is not to say that the choice of one retention system or the other lacked clinical significance. From a technical point of view, screw-retained prostheses offer a critical advantage in terms of retrievability. In patients at risk or with a history of previous malignance, screw-retained prosthesis facilitates the mandatory periodical check-up of the tissues underneath fixed rehabilitations [67, 68]. The same rationale can be applied to patients with soft tissue complications, in which screw-retention allows prosthetic removal and recontouring. [42]

Limited information on the performance of SI additively manufactured with other materials different from Ti alloys (PEEK or other materials) is available on the literature. As these materials could be considered very experimental, the group of 5 PEEK SI of the study of Mounir et al. [45] was excluded in the present review. Apart from the data of this study, a case series of 4 edentulous patients was published by Elsawy et al. [35] reporting survival of all the maxillary SI and no complications after a 12-month follow-up period. All the PEEK SI in their study had been manufactured with a 5-axis milling machine, therefore the study did not match the eligibility criteria of the present systematic review.

In summary, modern additively manufactured SI present good survival in the short-time but they still present a notable number of soft tissue complications. Comparing to traditional casted SI, soft-tissue complications could be probably more easily solvable (or containable in extension) as new CAD designs enables a simpler implant trimming and partial removing of the implant. This could reduce the influence of soft-tissue complications on implant survival. Nevertheless, the medium- or long-time clinical behavior is still to be clarified. They present several advantages over traditional casted ones. Better biocompatibility, one-time surgery possibility, a reduction in total mass of the material used, optimization of arms and fixation screws dimensions and number (thus reducing costs and avoiding micromovements) or time of surgery reduction (ensuring a better fitting and avoiding time to re-adapt bone), can be cited among the improvements [13–15]. New finite element method analysis on additively manufactured SI are desirable, to further enhance this advantages and also could be helpful to prevent overextending the implant.

In cases of extreme resorption, SI may be a feasible treatment option in the hands of experienced clinicians. However, in cases where residual bone available allows to room short root-shape implants or standard ones (even with the need for ancillary surgical procedures) the use of SI could be arguable as root-shape implants performance is further evidenced in the literature. Zygomatic implants are another alternative when the maxillary bone is completely or partially absent if the anatomy of the defect, the remnant bone and the maxillary sinus is favorable. However, zygomatic implants are also considered a complex treatment with significant surgical risk and potential for complications and the success of the treatment is highly dependent on the clinician experience. [69]

In sight of the results of the present study, the use of SI should be based on case selection such as severe atrophy and the impossibility (or unwillingness on the part of the patient) to conduct microvascular bone reconstruction or even patients that have a reduced expected lifespan.

A significant limitation of the present review is the absence of RCTs, prospective studies or other studies with a higher level of evidence in the available literature. A meta-analysis was not possible to obtain, due to this and the great heterogeneity between studies. On the other hand, no previous systematic review has been conducted on the topic to the best knowledge of the authors, and the results obtained could encourage to perform new well-designed studies to clarify the important lack of information in some key points for clinical practice. In sight of the results of the present systematic review, some treatment recommendations for former SI in older studies could be partially outdated.

## Conclusions

Subperiosteal implants have been used for decades, but lost relevance among clinicians due former poor clinical performance. Improvements through new technologies development have brought them to a new scenario. Based on the available studies (observational), “modern” CAD designed, and additively manufactured SI presented a satisfactory survival in the short time. However, further studies are needed to ascertain the success rate and the clinical behavior in the medium- and long-term. It would also be desirable to conduct further studies on CAD designed SI manufactured with the most modern subtractive manufacturing methods in view of the limited available clinical information.

Partial exposure was the most common complication reported. Post-operative complications, soft-tissue infection and interim prosthesis fracture were other remarkable complications reported. New SI designs may be helpful to prevent complications, but there is a need to strengthen the evidence with new clinical studies.

## Data Availability

The datasets used and/or analyzed during the current study are available from the corresponding author on reasonable request.
